# A Multi-Organ-on-Chip Approach to Investigate How Oral Exposure to Metals Can Cause Systemic Toxicity Leading to Langerhans Cell Activation in Skin

**DOI:** 10.3389/ftox.2021.824825

**Published:** 2022-02-15

**Authors:** Jasper J. Koning, Charlotte T. Rodrigues Neves, Katharina Schimek, Maria Thon, Sander W. Spiekstra, Taco Waaijman, Tanja D. de Gruijl, Susan Gibbs

**Affiliations:** ^1^ Department of Molecular Cell Biology and Immunology, Amsterdam University Medical Centre, Amsterdam Infection and Immunity Institute, Vrije Universiteit Amsterdam, Amsterdam, Netherlands; ^2^ TissUse GmbH, Berlin, Germany; ^3^ Department of Medical Oncology, Amsterdam University Medical Centre, Vrije Universiteit Amsterdam, Amsterdam, Netherlands; ^4^ Department of Oral Cell Biology, Academic Centre for Dentistry (ACTA), University of Amsterdam and Vrije Universiteit Amsterdam, Amsterdam, Netherlands

**Keywords:** organotypic, reconstructed human skin, reconstructed human gingiva, nickel, organ on a chip, Langerhans cell

## Abstract

Investigating systemic toxicity *in vitro* is still a huge challenge. Here, a multi-organ-on-chip approach is presented as a typical case of topical exposure of oral mucosa to metals, which are known to activate the immune system and in turn may result in skin inflammation. Reconstructed human gingiva (RHG) and reconstructed human skin containing MUTZ-3–derived Langerhans cells (MUTZ-LC) in the epidermis (RHS-LC) were incorporated into a HUMIMIC Chip3plus, connected by dynamic flow and cultured for a total period of 72 h. Three independent experiments were performed each with an intra-experiment replicate in order to assess the donor and technical variations. After an initial culture period of 24 h to achieve stable dynamic culture conditions, nickel sulfate was applied topically to RHG for 24 h, and LC activation (maturation and migration) was determined in RHS-LC after an additional 24 h incubation time. A stable dynamic culture of RHG and RHS-LC was achieved as indicated by the assessment of glucose uptake, lactate production, and lactate dehydrogenase release into the microfluidics compartment. Nickel exposure resulted in no major histological changes within RHG or RHS-LC, or cytokine release into the microfluidics compartment, but did result in an increased activation of LC as observed by the increased mRNA levels of CD1a, CD207, HLA-DR, and CD86 in the dermal compartment (hydrogel of RHS-LC (PCR)). This is the first study to describe systemic toxicity and immune cell activation in a multi-organ setting and can provide a framework for studying other organoids in the future.

## Introduction

All substances which come into contact with our body surfaces, for example, medical devices, drugs, and industrial-, agricultural-, and personal care products, have to be tested to determine whether they are safe for human exposure, and in most cases, to determine the lowest concentration which is safe for exposure. If humans are exposed to potentially sensitizing chemicals, they may develop allergic contact dermatitis in the form of skin eczema (rash). The adverse outcome pathway (AOP) for sensitization identifies the key events (KE) which could lead to such sensitization and allergy. In particular, KE-1 addresses chemical bioavailability, KE-2 addresses keratinocyte activation, KE-3 addresses dendritic cell (DC) activation, and KE-4 addresses T-cell priming (memory) (Rodrigues Neves and Gibbs, 2021).

Many *in vitro* methods which represent localized toxicological events within a single tissue or an organ have been described. For example, validated methods which integrate KE-1, KE-2, and KE-3 are now available to distinguish sensitizers from non-sensitizers and often to determine the sensitizer potency (Rodrigues Neves and Gibbs, 2021). The most promising of these *in vitro* assays using conventional submerged cell culture models are the KeratinoSens™ assay which represents activation of keratinocytes (KE-2) (Andreas et al., 2011), the genomic allergen rapid detection assay (GARD) (Johansson et al., 2019), the Myeloid U937 skin sensitization test (MUSST) (Ade et al., 2006), and the human cell line activation test h-CLAT (Ashikaga et al., 2006) which represents the activation of dendritic cells (KE-3). Advancements to more organotypic models make use of reconstructed human epidermis (RHE) and address KE-1 and KE-2 in a single assay. In the SENS-IS and EpiSensA assays, changes in gene expression are analyzed after a topical exposure of RHE to a chemical (Saito et al., 2013; Cottrez et al., 2015; Cottrez et al., 2016). In the RHE sensitizer potency assay, the release of IL-18 into cultured supernatants is used to identify sensitizers and also determine the potency of the sensitizer (Gibbs et al., 2013; Gibbs et al., 2018). The SenCeeTox assay uses RHE models to categorize chemical sensitizers by combining solubility, chemical reactivity, cytotoxicity, and activation of the Nrf2/ARE pathway (McKim et al., 2012). Notably, these models are all relatively simple, rely only on biomarker expression, and do not incorporate complex biology and immunotoxicity into a functional assay where one organ can influence another organ. Such a model would be required if systemic, rather than local toxicity is to be studied.

Therefore, the challenge is now to develop models and methods to investigate systemic toxicity, such as when a chemical breaches a body barrier and exerts a toxic event in a distant organ (Beilmann et al., 2019; Tavares et al., 2020; Picollet-D’hahan et al., 2021; Tao et al., 2021). The rapidly developing multi-disciplinary and interdisciplinary field of organ-on-chip (OoC) may provide the solution (Van den Broek et al., 2017; Heringa et al., 2020; Marx et al., 2020; Low et al., 2021; Picollet-D’hahan et al., 2021). A number of single OoC models which incorporate immune cells have been described. For example, lung alveolus models which incorporate flowing immune cells (macrophages, neutrophils, or peripheral blood–derived monocytes (PBMC)) below a co-culture of endothelial and lung epithelium cells have been described. Cell–cell interactions, dynamic flows, and stretches of the Human Emulate System^®^ improve the functionality of the lung model, resulting in *in vivo*–like cell differentiation, cilia function, mucociliary clearance, and a tight epithelial barrier (Huh et al., 2010; Benam et al., 2016; Thacker et al., 2020). Similar barrier models have also been described to investigate host–microbiome interactions in the gut using as an epithelial cell source, the cell line Caco-2, immune cells such as PBMC, neutrophils, U937, and THP-1, and a variety of relevant gut bacteria (Ramadan and Jing, 2016; Maurer et al., 2019; Gjorevski et al., 2020). Also, a number of multi-OoC models have been described, which also incorporate the skin. For example, 2-OoC models include combinations of preformed skin or lung models with human liver spheroids (Maschmeyer et al., 2015a; Schimek et al., 2020), and 4-OoC models include a preformed skin and human intestine being co-cultured with liver spheroids and human proximal tubule epithelial cells (kidney) (Wagner et al., 2013; Maschmeyer et al., 2015b). These multi-OoC models implement a dynamic circulation with vascular perfusion within a closed circuit using HUMIMIC chips (TissUse, Berlin, Germany) which enable perfusion of organoids and spheroids under physiologically relevant shear stress and tissue to fluid ratios.

Notably, no multi-organ models which also integrate immune cells have yet been described. This is required if systemic immunotoxicity events are to be studied. For example, currently used dental biomaterials have been shown to leach degradation products into the oral cavity in patients. This may lead to leachables or other toxicants gaining access to the circulation and accumulation in major organs (e.g., the liver, heart, brain, and even skin), resulting in systemic toxicity in these organs (Aquino and Mucci, 2013; Roos et al., 2013; Matusiewicz, 2014). Indeed, in susceptible individuals, it has been shown that fixed metallic retainer wires in the oral cavity can lead to chronic facial eczema, which remains visible for up to 2 months after the removal of the device even when no signs of oral mucosa inflammation are observed (Feilzer et al., 2008). One explanation for this phenomenon is systemic exposure to toxic nickel ions, which are known to be skin sensitizers, accumulating in the skin resulting in eczema, with tolerance to these same metal ions occurring in the oral mucosa (Van Hoogstraten et al., 1993; Muris et al., 2015).

Previously, we have described reconstructed human gingiva (RHG) and skin (RHS) models with integrated MUTZ-3-derived Langerhans cells (MUTZ-LC) to investigate localized immune events involved in tolerance, sensitization, and elicitation of contact allergy (Ouwehand et al., 2011; Kosten et al., 2015a; Kosten et al., 2016). These models consist of a differentiated and stratified epithelium with or without integrated MUTZ-LC on a fibroblast-populated collagen hydrogel which is cultured with exposure to air from above with nutrients from the culture medium being supplied *via* contact with the hydrogel from below. These models permit LC plasticity and migration from the epidermis to the hydrogel (dermis), thus providing a unique immunotoxicological research tool for investigating chemical allergens and irritants. The MUTZ-LCs were fully functional and closely resembled their *in vivo* LC counterpart (Masterson et al., 2002; Larsson et al., 2006; Santegoets et al., 2006).

The aim of this study was to present a new method for investigating systemic immunotoxicity in a multi-organ-on-chip setting. The microfluidics bioreactor is a HUMIMIC Chip3plus (TissUse, Berlin Germany) which enables stable dynamic flow within a closed circuit (Schimek et al., 2020). Here, we determine whether leachables from dental restorative materials, for example, nickel ions, when applied to RHG can cause the elicitation of an extra-oral innate immune response in RHS with integrated MUTZ-LC cells (RHS-LC). Attention is paid to inter- and intra-experimental reproducibility and stability of the dynamic flow culture system over a period of 72 h (glucose uptake, lactate production, and lactate dehydrogenase (LDH) release as well as MUTZ-LC phenotype and migration from the epidermis to the dermis hydrogel and inflammatory cytokine release).

## Materials and Methods

### Isolation and Culture of Human Skin and Gingiva Keratinocytes and Fibroblasts

Human skin and gingiva were obtained as a surgical waste after getting informed consent from healthy donors who underwent an abdominal dermolipectomy or dental implant surgery. Both the skin and gingiva were used in an anonymous fashion in accordance with the “Human Tissue and Medical Research: Code of conduct for responsible use” as formulated by the Federation of Dutch Medical Scientific Societies (www.federa.org).

Skin and gingiva keratinocytes were isolated and cultured as described previously (Waaijman et al., 2010). Epithelial sheets were separated from the dermis (skin) or lamina propria (gingiva) by an overnight incubation in dispase II (Sigma-Aldrich, St. Louis, MO, United States) at 4°C. The epithelial sheet was then trypsinized (Trypsin; Gibco, Grand Island, United States), and the primary keratinocytes were cultured at 37°C and 7.5% CO_2_ in a KC medium consisting of Dulbecco’s modified Eagle medium (DMEM; Lonza, Basel, Switzerland)/Ham’s F-12 (Gibco) (3:1), 1% Ultroser G (BioSepra S.A. Cergy-Saint-Christophe, France), 1% penicillin–streptomycin (Gibco), 1 μM hydrocortisone (Sigma-Aldrich), 1 μM isoproterenol (Sigma-Aldrich), and 0.1 μM insulin (Sigma-Aldrich), and supplemented with 1 ng/ml keratinocyte growth factor (KGF; Sigma-Aldrich) for skin keratinocytes or 1 ng/ml epidermal growth factor (EGF; Sigma-Aldrich) for gingival keratinocytes. This culture medium, containing 1.6 mM calcium, enables keratinocytes to differentiate as well as proliferate. The keratinocytes were used at passage 1.

Skin and gingival fibroblasts were isolated from the dermis/lamina propria (after the removal of the epithelium) by incubation in dispase with collagenase (Gibco) for 2 h. After passing the suspension through a 40 μm cell strainer (Corning Life Science, United States), to remove extracellular matrix, the fibroblasts were cultured at 37°C, 5% CO_2_ in a fibroblast medium containing DMEM, 1% Ultroser G, and 1% penicillin–streptomycin. Fibroblasts were used at passage 1 or 2.

### MUTZ-3 Cell Culture

MUTZ‐3 progenitor cells (Deutsche Sammlung von Mikroorganismen und Zellkulturen, Braunschweig, Germany) were maintained as previously described (Masterson et al., 2002) until maximum passage number 35. MUTZ‐3 progenitor cells were differentiated into LCs (MUTZ‐LC) in minimal essential medium‐alpha (Gibco) supplemented with 20% vol/vol heat‐inactivated calf serum (HyClone laboratories, Logan, Utah), 1% penicillin–streptomycin, 2mM l‐glutamine (Gibco), 50  μM 2‐mercaptoethanol (Merck, Whitehouse Station, New York), 100 ng/ml recombinant human granulocyte macrophage–colony stimulating factor (GM-CSF; BioSource International, Camarillo, California), 10 ng/ml TGF‐β, and 2.5 ng/ml TNF‐α (both R&D systems, Minneapolis, Minnesota) for 7 days at 37°C, 5% CO_2_, and 95% humidity. MUTZ‐LCs were labeled with carboxyfluorescein succinimidyl ester (CFSE; Thermo Fisher, Waltham, Massachusetts) before incorporation into the RHS as described previously (Ouwehand et al., 2010).

### Reconstructed Human Skin With Incorporated MUTZ-Derived Langerhans Cells and Reconstructed Human Gingiva

RHS-LC and RHG were constructed in 12 mm 0.4 µm MilliCell standing cell culture inserts (Merck Millipore Ltd., Tullagreen, Cork, Ireland) in 24 well plates. Dermal equivalents were first constructed by mixing rat tail collagen with fibrinogen (Enzyme Research Laboratories, South Bend IN, United States) at a ratio of 4:1 in Hank’s Balanced Salt Solution (HBBS; Gibco, final concentration collagen is 4 mg/ml and fibrinogen is 1 mg/ml), and dermal fibroblasts (2.5 × 10^4^ cells/gel) were then added to the solution on ice. Thrombin (0.5 U/ml, Merck KGaA, Darmstadt, Germany) was added to each hydrogel to allow fibrin formation, and gels were pipetted into cell culture inserts. Hydrogels were incubated for approximately 30 min at 37°C and 7.5% CO_2_ to promote polymerization, and then submerged in the KC medium. After 24–48 h, keratinocytes were seeded onto the fibroblast-populated hydrogels at a density of 6.5 × 10^4^ cells/gel. RHS-LC models were created by seeding 1.3  ×  10^5^ CFSE-labeled MUTZ-LCs/gel onto the dermal equivalents 2 h prior to keratinocyte seeding. RHG and RHS-LC were cultured under submerged conditions for 3 days in KC1 medium supplemented with 1 ng/ml KGF (RHS-LC) or 1 ng/ml EGF (RHG) at 37°C and 7.5% CO_2_. Before being transferred to the HUMIMIC Chip3plus (TissUse, GmbH, Berlin, Germany), constructs were lifted to the air–liquid interface for 8 days and cultured in the KC2 medium consisting of Dulbecco’s modified Eagle medium (DMEM)/Ham’s F-12 (3:1), 0.2% Ultroser G, 1% penicillin–streptomycin, 1 μM hydrocortisone, 1 μM isoproterenol, and 0.1 μM insulin supplemented with 50 ug/ml vit C and 2 ng/ml KGF (RHS-LC) or 2 ng/ml EGF (RHG) at 37°C and 7.5% CO_2_.

### RHG and RHS-LC Culture in HUMIMIC Chip3plus

**FIGURE 1:**
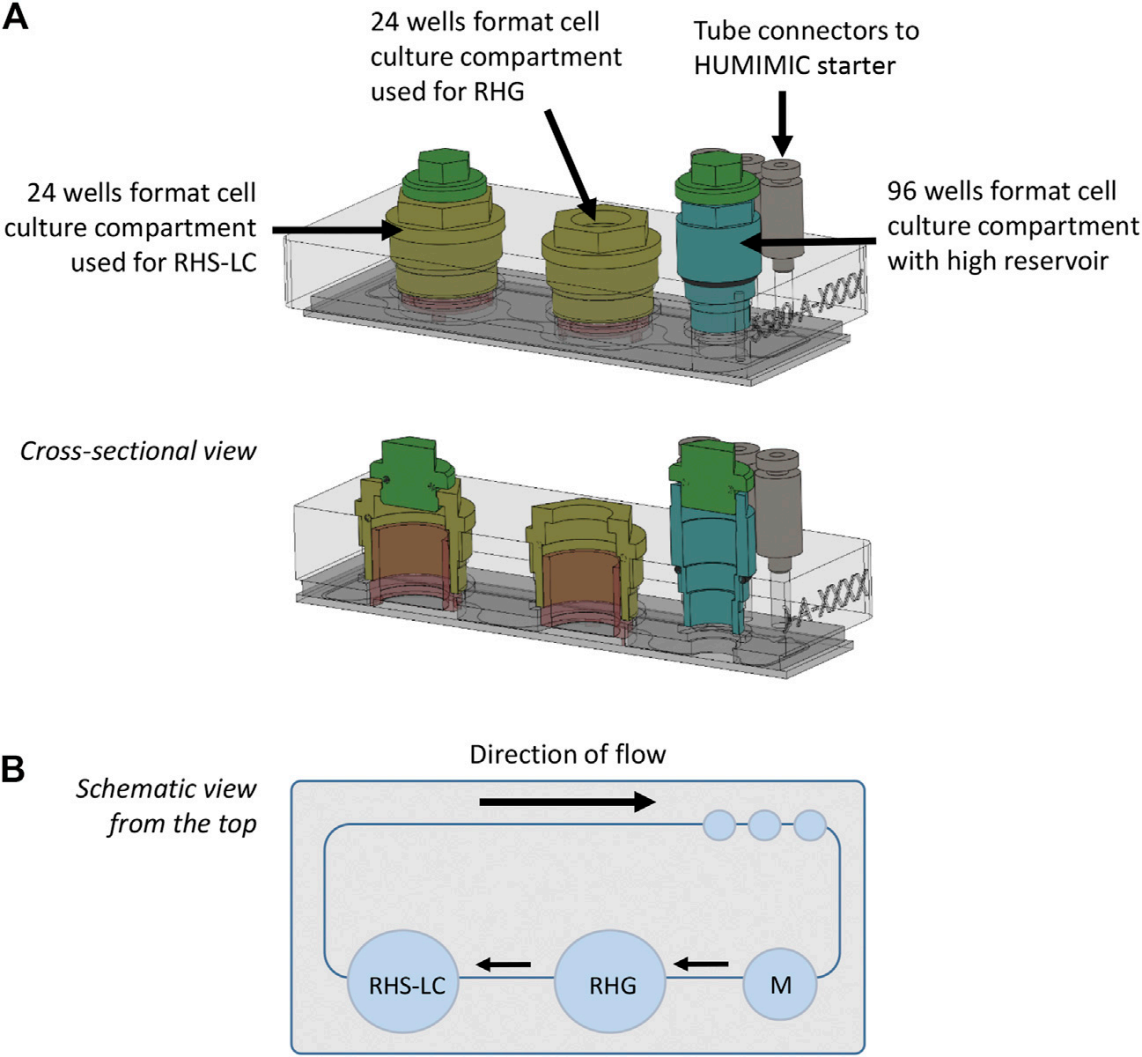
HUMIMIC Chip3plus overview and experimental setup. **(A)** Schematic illustration of the HUMIMIC Chip3plus including the loading scheme of the relevant organ equivalents. **(B)** Schematic top view of the multi-organ chip; arrows indicate direction of flow. RHS-LC, reconstructed human skin with Langerhans cells; RHG, reconstructed human gingiva; and M, medium.

The HUMIMIC Chip3plus contains three consecutive culture compartments (2 × 24 well format and 1 × 96 well format) connected by microfluidic channels (Figure 1A (Schimek et al., 2020)). An external control unit (HUMIMIC starter, TissUse) regulates fluid flow in the Chip3plus. RHG and RHS-LC were co-cultured in 24 well culture compartments and cultured under a pulsatile flow for a total of 3 days (72 h) at 37°C, 7.5% CO_2_. The 96 well cell culture compartment with high reservoir was used for medium exchanges. The culture medium consisted of the KC2 medium without hydrocortisone and supplemented with 50 ug/ml vit C, 2 ng/ml KGF, and 2 ng/ml EGF. RHG were cultured with 200 µl medium underneath the culture, RHS-LC were cultured with 400 µl medium underneath the culture, and 700 µl medium was added to the medium reservoir. Both RHG and RHS-LC were further cultured at the air–liquid interface. The compartment containing RHG was sealed with a gas-permeable cellulose fiber–based membrane (non-woven white Rayon, Corning) that allows sterile culture of the construct at the air–liquid interface while at the same time enabling its easy removal to apply the test substances (vehicle and nickel sulfate). Both other compartments were closed with a standard, removable lid. Chips were connected to the HUMIMIC starter to create a pulsatile flow at 0.5 Hz and 500 mBar (50 kPa). Flow direction is indicated in Figure 1B. Every 24 h, 500 µl culture medium within the chip was exchanged with a new medium *via* the 96 well cell culture compartment, and used for the analysis of LDH, lactose, glucose, cytokine secretion, and nickel ion concentration. After 3 days of dynamic flow in the HUMIMIC Chip3plus, RHG and RHS-LC were harvested for analysis. Each replicate was divided in parts for histology, RNA isolation, and epidermal sheet imaging.

### Exposure of RHG to NiSO_4_

After 24 h in the HUMIMIC Chip3plus, RHG were exposed to the sensitizer NiSO_4_ (10 mM) or its vehicle H_2_O by impregnating 03-150/38 gauze filters of 6 mm diameter (Sefar Nitex, Heiden, Switzerland) as described previously (Gibbs et al., 2013). The impregnated gauze filters were applied topically to the stratum corneum of RHG for 24 h. After 24 h, gauze filters were gently removed, and cultures were left for another 24 h in the HUMIMIC Chip3plus (resulting in a total of 3 days, 72 h).

### Assessment of Glucose, Lactate, and LDH

The culture supernatant (500 µl sample of the total 1,300 µl volume) derived from the co-culture of RHG and RHS-LC in the HUMIMIC Chip3plus was harvested at day 1, day 2, and day 3 of the dynamic cell culture. This was replenished with 500 µl of the fresh KC2 medium. Glucose, lactate, and LDH amounts were determined in the sample as described by the kit suppliers using the Indiko Plus system (Thermo Fisher Scientific, Germany) in combination with the Glucose (HK) 981779 kit (Thermo Fisher Diagnostics GmbH, Germany), Lactate Fluitest LA 3011 kit (Analyticon Biotechnologies AG, Germany), and LDH IFCC 981782 kit (Thermo Fisher Diagnostics GmbH). All absorbance measurements were performed in 96 well microtiter plates (Greiner Bio-One, Frickenhausen, Germany).

### Histology and Immunohistochemical Staining

RHG and RHS‐LCs were fixed in 4% formaldehyde and embedded in paraffin. Paraffin sections (5 μm) were used for morphological (hematoxylin and eosin staining) and immunohistochemical analysis of Loricrin (clone AF62, Covance, Emeryville CA, United States) and K10 (clone DE-K10, Progen, Heidelberg, Germany). Antigen retrieval was performed using a citrate buffer (0.01 M, pH 6) and subsequent pepsin incubation for K10 staining. For Loricrin staining, endogenous peroxidase was blocked with 0.3% H_2_O_2_ in a methanol solution and blocked with goat serum prior to antibody incubation. Sections were incubated overnight with primary antibodies, and after washing, they were incubated with anti–mouse-conjugated HRP (K10) or anti–rabbit-conjugated HRP (Loricrin). Sections were subsequently incubated with 3-amino-9-ethylcarbazole to visualize antibody staining. All sections were counter-stained with hematoxylin and embedded in Aquatex (Merck Millipore, Germany). Tissue sections were photographed using a Nikon Eclipse 80i microscope (Düsseldorf, Germany) with NIS elements AR 3.2 software (Nikon, Tokyo, Japan).

### mRNA Transcript Analysis

Epidermal sheets from RHS-LC were separated from the dermal hydrogel using forceps. The epidermis was used to image LC in the sheet, and the hydrogel was snap frozen at −80°C. From the hydrogel, RNA was isolated using an RNeasy Mini Kit (Qiagen, Hilden, Germany) and cDNA was synthesized using a RT2 First Strand Kit (Qiagen), all according to the manufacturer’s instructions. Quantitative RT-PCR was performed on a ViiA7 Real-Time PCR machine or a StepOnePlus Real-Time PCR System (both ThermoFisher Scientific, United States). The total volume of the reaction mixture was 10 μl, containing cDNA, 300 nM of each primer, and SYBR Green Mastermix (PE Applied Biosystems). The following primers (OriGene) were used: CD1a (hp205560), CD86 (hp233662), GAPDH (hp205798), and HLA-DRB1 (hp205865). The comparative Ct method (∆∆Ct) with GAPDH as the housekeeping gene was used to indicate relative changes in mRNA levels between samples. Relative mRNA levels of control-treated tissues were set at 1.0.

### Determination of Nickel Concentration

The nickel concentration in culture supernatants was determined by graphite furnace atomic absorption spectrometry (GFAAS; PinAAcle 900Z, PerkinElmer, Singapore) using a hollow cathode lamp. Samples were acidified with nitric acid (Fisher scientific, Optima Grade) before analysis. Each sample was measured in triplicate.

### Cytokine Detection in Culture Supernatants

Frozen supernatants were thawed and analyzed for cytokines using an inflammatory cytokine bead array (CBA; BD biosciences), according to the manufacturer’s instructions with slight modifications. Acquisition was performed on a BD LSR Fortessa Flow Cytometer (BD Biosciences). Analysis was performed in FCS Express v7 (De Novo Software, United States). Secretion of IL-18 was measured using an enzyme-linked immunosorbent assay (ELISA; MBL International, Woburn, MA, United States).

### Imaging Epidermal Sheets

Epidermal sheets were incubated with CD83 antibodies (BD Pharmingen, San Diego, United States) for 1 h and washed with PBS. CFSE- and CD83-labeled MUTZ-LC were visualized using a Keyence fluorescence microscope (Osaka, Japan).

### Data Analysis

For RHS-LC, keratinocytes and fibroblasts from the same skin donor were used. For RHG, keratinocytes and fibroblasts were obtained from different donors. RHS-LC and RHG were not donor-matched; separate skin and gingiva donors were used for each independent experiment, and cells from different donors were never pooled.

In total, three independent experiments were performed. For the first experiment, 3 RHG and 3 RHS-LC were constructed to enable 3 intra-experiment replicates, and for the second and third experiments, 2 RHG and 2 RHS-LC were constructed to enable 2 intra-experiment replicates. All cultures were analyzed for histology and mRNA expression, and all supernatants were analyzed for glucose, lactate, LDH, nickel concentration, and cytokine secretion.

All data are presented as mean ± standard error of the mean (SEM). Statistical analysis was performed by means of paired *t*-test and 2-way ANOVA followed by Bonferroni’s multiple comparisons test using GraphPad Prism 9 software (GraphPad Software Inc., La Jolla, United States) as indicated in figure legends. Differences were considered to be significant when *p* < 0.05.

## Results

### Establishing Stable Dynamic Flow of RHG and RHS-LC

**FIGURE 2:**
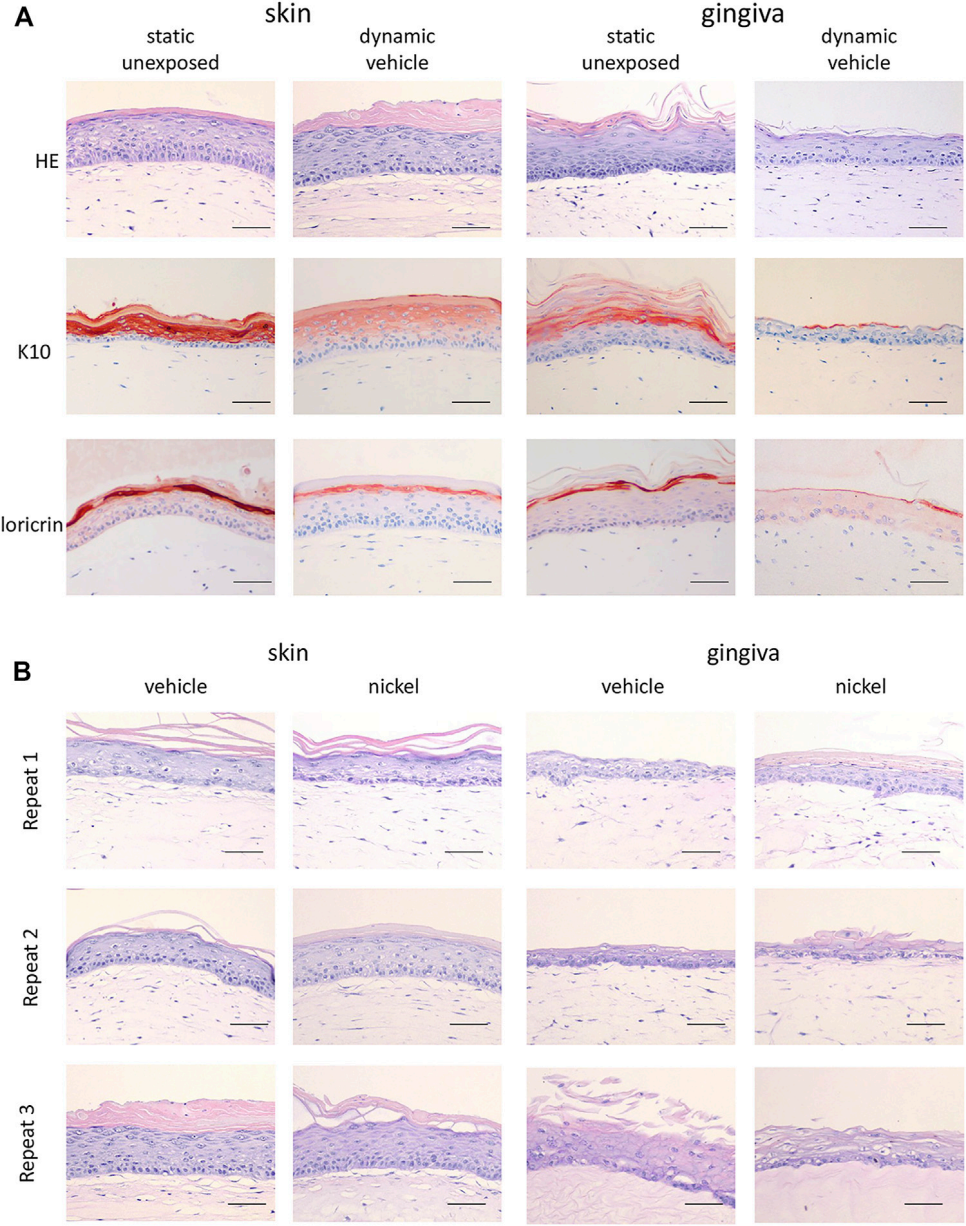
Histological analysis of RHS-LC and RHG. **(A)** Hematoxylin and eosin (H&E), K10, and Loricrin staining of paraffin-embedded tissue sections (5 μm) to visualize histology of RHG as well as RHS-LC in static cultures as well as dynamic cultures. **(B)** H&E staining of three experimental independent co-cultures of RHG and RHS-LC showing histology after exposure to either the vehicle or nickel sulfate. The scale bar represents 50 µm (A and B).

In line with our previous publications, the static RHS-LC and RHG prior to incorporation into the HUMIMIC Chip3plus consisted of a stratified and differentiated epithelium on a fibroblast-populated hydrogel which represented the dermis and lamina propria, respectively (Kosten et al., 2015a; Kosten et al., 2015b). The differential expression of keratin 10 and Loricrin confirmed the skin and gingiva phenotypes of the organotypic models with RHS-LC, showing consistent suprabasal K10 expression and Loricrin confined to the granular layer, and RHG, showing intermittent K10 and lower Loricrin expression (Figure 2A). When RHG and RHS-LC were incorporated into the HUMIMIC Chip3plus, RHG were topically exposed to H_2_O (vehicle for NiSO_4_), both RHS-LC and RHG were further cultured under dynamic flow conditions for 3 days, and these phenotypes were maintained (Figure 2A). However, the uppermost cell layers of RHG were removed together with the vehicle-impregnated gauze filter, resulting in a slightly thinner RHG than those observed under static conditions. Within the 3 replicate experiments performed under dynamic flow, very little inter-experiment phenotypic variation was observed, even after topical exposure of RHG to NiSO_4_ (Figure 2B).

**FIGURE 3:**
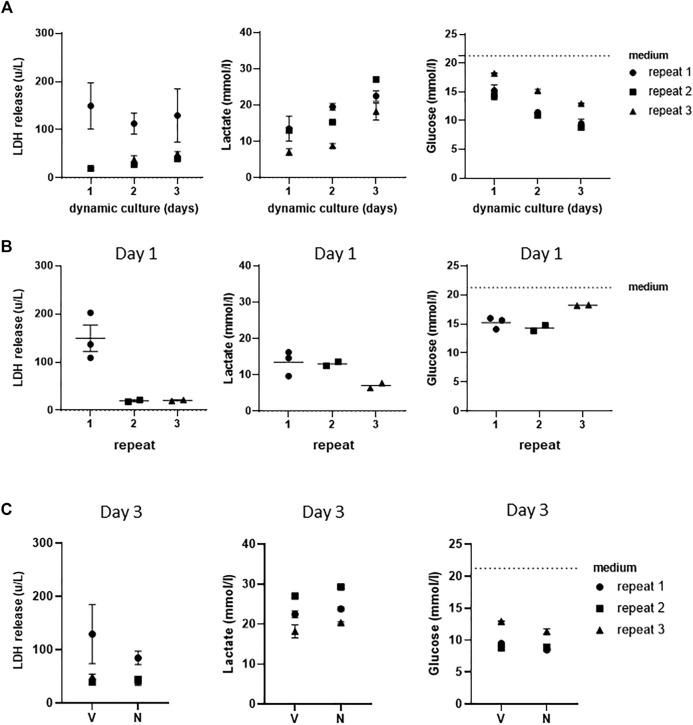
Assessment of stable co-cultures under dynamic flow for 3 days. **(A)** LDH, lactate, and glucose measurements of three independent experiments showing achievement of stable cultures over 3 days of culturing under dynamic flow. **(B)** Assessment of intra- and inter-experimental variation of LDH release, lactate, and glucose consumption at day 1. **(C)** Levels of LDH, lactate, and glucose in supernatants of vehicle (V)- and NiSO_4_ (N)-exposed co-cultures at day 3. The data represent mean ± SEM; n = 3.

In order to further investigate whether a stable culture under dynamic flow conditions had been achieved, LDH release, which is indicative of porous cell membranes and cytotoxicity, was determined and found to remain unaltered in vehicle-exposed co-cultures during the 3 days of culture (Figure 3A). When cells in culture are actively metabolizing, they produce lactate and take up glucose from the culture medium. During the 3 days of dynamic flow, lactate concentrations doubled, and glucose concentrations halved in the culture medium as a further indication that stable dynamic culture had been achieved (Figure 3A).

Next, the intra- and inter-experimental variations were determined. An extremely little intra-experimental variation was observed with regard to LDH, lactate production, and glucose uptake in vehicle-exposed co-cultures (Figure 3B). Some variation was observed between the different repeat experiments, with LDH release being higher in the first experiment than that in the second and third experiments. Furthermore, lactate production was lower in the third experiment, which correlated to a slightly lower glucose uptake in the same replicate experiment. Although Figure 3B only shows results after 1 day of dynamic flow, similar results were also observed on day 2 and day 3 samples (data not shown).

### Nickel Ions Detected in Culture Medium After Topical NiSO_4_ Application to RHG

**FIGURE 4:**
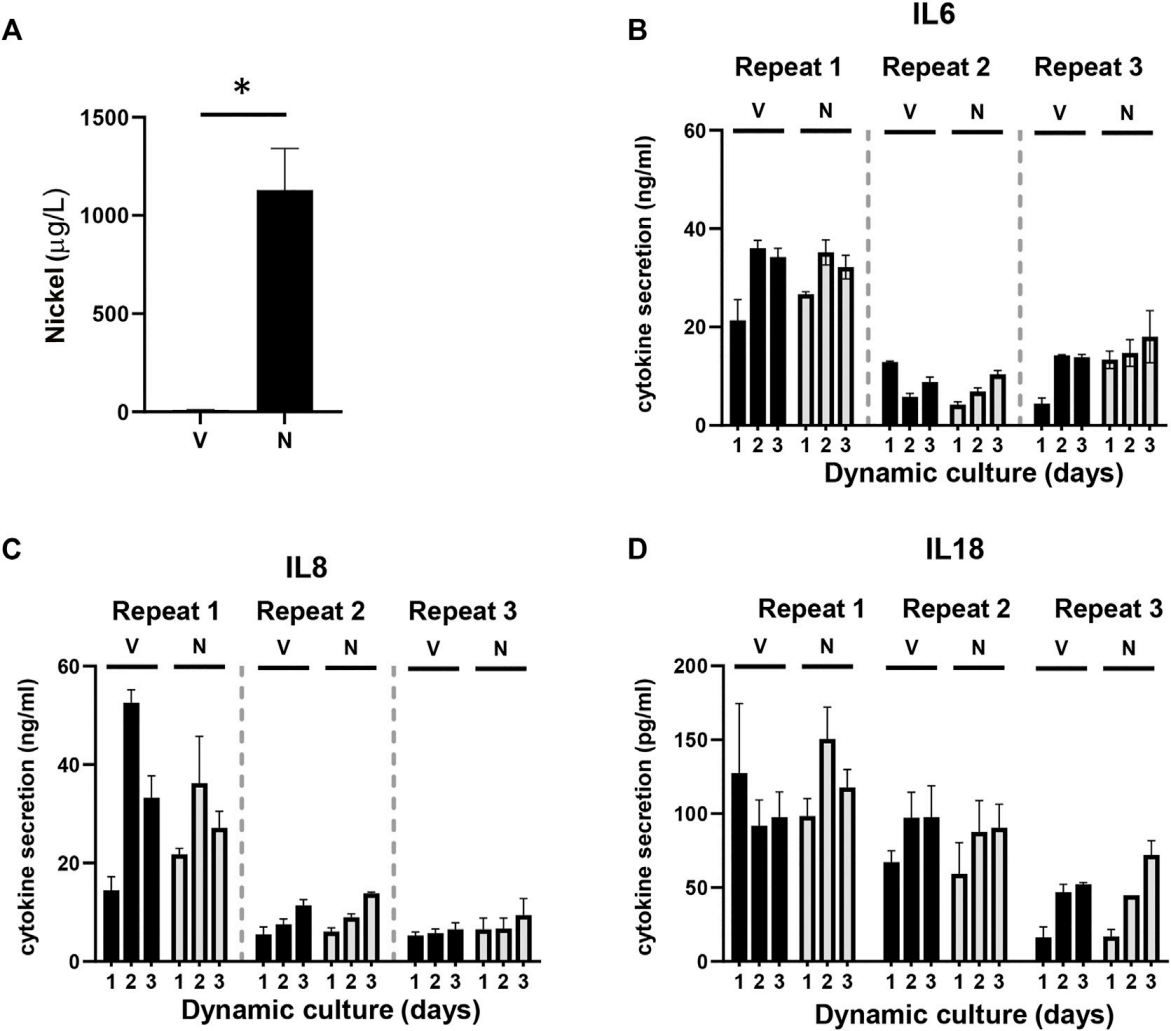
Detection of nickel ions and interleukins. **(A)** Detection of the amount of nickel ions in culture supernatants derived from HUMIMIC Chip3plus, measured with graphite furnace atomic absorption spectrometry (GFAAS) in both vehicle (V)- and nickel (N)-exposed co-cultures at day 3. (B-D) Quantification of IL-6 (B), IL-8 **(C)**, and IL-18 **(D)** in supernatants of multi-organ cultures upon exposure to the vehicle (V) or NiSO4 (N) over 3 days of culturing. The data represent mean ± SEM; n = 3. *, *p* < 0.05; paired *t*-test (A), 2-way ANOVA followed by Bonferroni’s multiple comparison test **(B–D)**.

Since the aim of this study was to establish a model for systemic toxicity, we topically exposed RHG to NiSO_4_ (10 mM) at day 1 of culturing in the HUMIMIC Chip3plus, removed the NiSO_4_ gauze patch at day 2, and determined whether nickel ions were able to penetrate RHG and leach into the circulating culture medium at day 3. Graphite furnace atomic absorption spectrometry (GFAAS) detected a concentration of 1,079 μg/L (±225 μg/L, SEM) of nickel ions in the circulating culture medium beneath the RHG and RHS-LC co-cultures (Figure 4A). These nickel ions were not cytotoxic to either RHG or RHS-LC as indicated by no difference in LDH release, lactate production, or glucose uptake compared to vehicle-exposed RHG (Figure 3C). Negligible amounts of nickel ions were detected in vehicle-exposed co-cultures.

### Topical NiSO_4_ Application to RHG Does Not Result in an Increased Cytokine Release

Next, it was determined whether the nickel ions were able to trigger cytokine release from the RHG and RHS-LC co-culture models by assessing cytokines within the circulating culture supernatant. Expression of immune suppressive (IL-10 and IL-12p70) and (pro) inflammatory cytokines (IL-8, IL-1β, IL-6, and TNF-α) was determined with a cytokine bead array, and IL-18 levels were determined by Elisa. IL-6, IL-8, and IL-18 were detected in culture supernatants, but no differences in cytokine levels were observed between the vehicle- and nickel-exposed cultures. Cytokine levels had the tendency to increase slightly at day 2 and day 3 compared to those in day 1 samples (Figures 4B–D). The intra-experimental variation was low; however, lower cytokine levels were observed in experiment 2 and 3 than those in experiment 1. The other cytokines were below the detection limit of the assay. Taken together, these data indicate low levels of (pro) inflammatory cytokine release into the circulation upon topical exposure of RHG to NiSO_4_.

### Topical Application of NiSO_4_ to RHG Results in LC Activation in RHS-LC

**FIGURE 5:**
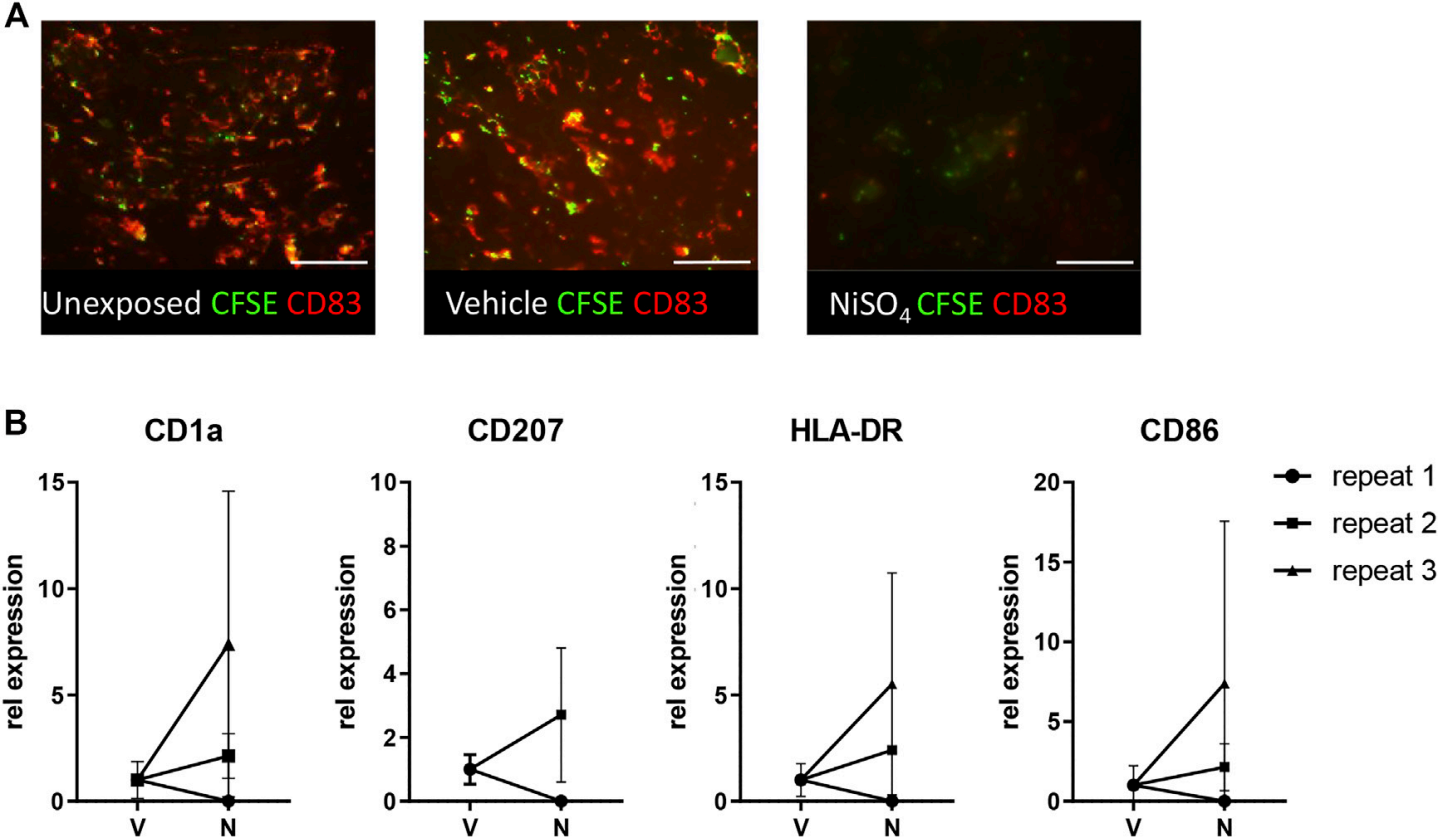
Topical application of NiSO_4_ to RHG results in LC activation in RHS-LCs. **(A)** Immunofluorescence analysis of epidermal sheets derived from RHS-LC with Langerhans cells (LC) labeled with CFSE (green) and CD83 (red) from unexposed, vehicle-exposed (24h +24 h), or NiSO_4_-exposed (24h +24 h) cultures. The scale bar represents 100 µm. **(B)** mRNA expression levels of LC markers in the dermis of RHS-LC relative to a housekeeping gene showing intra- and inter-experimental variation. CD207 expression for repeat experiment 3 was eliminated from results due to the CT level being above the threshold. The data represent mean ± SEM; n = 3. *, *p* < 0.05; paired *t*-test (A).

Next, it was determined whether the circulating nickel ions could activate the LC within the epidermis (Figure 5). Imaging of the epidermal sheets showed that in vehicle-exposed RHS-LC harvested 3 days after its incorporation into the HUMIMIC Chip3plus, the CFSE^+^ CD83^+^ LC were distributed evenly throughout the epidermis at a similar density to LC in the unexposed RHS-LC before incorporation into the HUMIMIC Chip3plus. After NiSO_4_ exposure to RHG, MUTZ-LC levels were reduced (repeat exp 1, data not shown) or no longer detected (repeat exp 2 and 3) in RHS-LC (Figure 5A). To determine whether the LC had become activated and migrated into the fibroblast-populated hydrogel, mRNA isolated from the hydrogel was assessed for CD1a, CD207 (langerin), HLA-DR, and CD86 (Figure 5B). Although repeat experiment 1 did not show this LC activation, repeat experiments 2 and 3 showed an increase in CD1a, CD207, and HLA-DR mRNA levels, indicating migration of LC into the hydrogel and also an increase in CD86 mRNA which indicates maturation of LC in the hydrogel after NiSO_4_ exposure.

## Discussion

This technical advance describes a method to investigate an immune event triggered by a distant organ in a multi-organ-on-chip setting which presents a relevant clinical observation.

We were able to show that an oral exposure (RHG) to NiSO4 resulted in nickel ions leaching into the dynamic flow compartment of the HUMIMIC Chip3plus, where they then activated RHS-LC resulting in LC maturation and migration out of the epidermis and into the dermis hydrogel. This study is the first of its kind to represent systemic immunotoxicity between barrier organs. The co-culture of RHS-LC and RHG in the HUMIMIC Chip3plus enabled cytokine secretion, metabolism, and cytotoxicity to be investigated on a daily basis for 3 days by sampling from the closed circuit dynamic flow compartment.

In this study, we paid particular attention to intra- and inter-experimental variations since this is an absolute requirement when developing robust methods. Therefore, three independent repeat experiments were performed each with an intra-experiment replicate in order to assess donor and technical variation. Notably, whereas intra-experiment replicates showed very little variation, variation was observed between repeat experiment 1 and the other 2 experiments. Repeat experiments 2 and 3 showed LC migration out of epidermal sheets and an increase in mRNA levels in hydrogel indicative of LC migration and maturation (CD1a, CD207 (langerin), HLA-DR, and CD86). This was not observed for repeat experiment 1. Furthermore, repeat experiment 1 showed higher LDH release and cytokine levels (IL-6 and IL-8) than repeat experiments 2 and 3, whereas lactate and glucose concentrations, as well as histology, were in line with the other repeats, and therefore, the differences could not be simply due to differences in the quality of the RHG and RHS-LC which would, in turn, influence how the co-culture would respond to the nickel ions. Whether the differences observed between repeat experiment 1 and the other 2 repeat experiments is due to donor variation (different skin and gingiva donors were used in each experiment) or technical variation cannot be determined at this stage. If the differences were indeed due to donor variation, this would be extremely interesting as it might explain in part why one individual responds with an allergic skin reaction to oral exposure of nickel whereas another does not (Feilzer et al., 2008). In the past, sensitization has been linked with the level of DC activation which has to be above a certain threshold level to initiate a T-cell response (Seneschal et al., 2012).

In this study, we assessed nickel ion concentrations as the proof of principle that in our model, nickel ions can penetrate through the RHG and into the circulation as one would expect to occur in humans. Nickel ion concentrations were only measured at day 3, which is the end of the experiment. However, both at day 1 and day 2, 500 µl of supernatants were harvested for quality assessment and for the measurement of cytokines, and subsequently replaced with a new medium, and therefore, the measured concentration at day 3 is an underestimation of the total amount of nickel ions that passed through the RHG. Because of low volumes of culture supernatants harvested at day 1 and day 2 (appr. 500 µl), we were not able to measure nickel ion concentrations at these timepoints, in order to correct for the total amount (in addition to other markers). Having said that, we do show that 18.4 µM (1,079 μg/L) of nickel ions were detected in the culture supernatant at day 3. Previously, we have shown that 250 µM of nickel chloride was sufficient to increase IL-8 secretion from pure primary keratinocytes cultured under conventional submerged culture conditions (Rachmawati et al., 2015) and that 150 µM Nickel chloride was sufficient to stimulate pure MUTZ-LC migration and IL-8 release in our dendritic cell migration assay (Ouwehand et al., 2010). Therefore, although the amount of nickel ions detected in the circulating culture medium beneath the RHG and RHS-LC co-culture is low, we do consider that within the microenvironment created within the RHS-LC model it should be sufficient to stimulate MUTZ-LC migration and activation. Indeed, this low amount could be around threshold levels required for activation and might explain why only repeat experiments 2 and 3, but not 1, showed LC activation. Of note, very high chemical concentrations (10 mM) are required only for topical exposures when penetration of a barrier is required such as with the RHG-differentiated epithelium and stratum corneum. Submerged or systemic exposure requires much lower chemical concentrations to activate cells directly without a barrier.

Due to the small size of the constructs as well as the low volume of the culture medium (1,300 µl) used in the HUMIMIC Chip3plus, the number of readouts was limited. Quality assessment of the constructs requires at least basic histology (H&E staining) and analysis of supernatants for LDH, glucose, and lactate, thereby leaving less material available for the assessment of other parameters. In order to better assess the migration of LCs from the epidermis into the dermis, future studies should include flow cytometric analysis of both epidermal sheets and hydrogels which would allow for better phenotyping and quantification of the amount of LCs. This requires reproducible isolation methods of viable cells from the constructs. Additional spatial information on migrated LCs would require 3D imaging of large parts of the constructs which is possible with light sheet microscopy, although not at a high throughput level yet.

Using cytometric bead arrays, we were able to analyze various inflammatory parameters in culture supernatants using small volumes. We were unable to determine if detected cytokines were released from RHG or RHS-LC. This requires either extraction of mRNA from the constructs for RT-PCR analysis or extraction of proteins from tissue lysates for cytokine detection by ELISA or a cytometric bead array. Although we cannot determine whether cytokines were released from RHG or RHS-LC, it is likely to assume that direct exposure of RHG to nickel sulfate would result in stronger activation of the construct when compared to RHS-LC, which only comes in contact with nickel ions *via* the circulation. However, exposure to nickel sulfate did not result in increased levels of pro-inflammatory cytokines IL-6, IL-8, and IL-18. For IL-18, this is in-line with previous research where we show that metal salts do not result in increased IL-18 levels in reconstructed human epidermis (RHE, (Gibbs et al., 2018)) and RHG (Shang et al., 2020). Notably, RHG and RHS are able to produce increased levels of general inflammatory mediators IL-6 and IL-8 upon exposure to nickel sulfate when compared to the vehicle (Kosten et al., 2015b; Shang et al., 2020) although higher concentrations of nickel sulfate were applied previously than those in this study (20 vs 10 mM, respectively). Although in this study we did not include the assessment of pro-inflammatory cytokines, it is most probable that these are released at low concentrations within the RHG and RHS-LC and immediately removed from the circulating culture supernatant by the keratinocyte and fibroblasts within the tissue constructs. A future study will be extended with mRNA analysis of the RHG and RHS-LC to clarify this. Despite similar cytokine levels between the 2 conditions (Figure 4), we did observe migration of LCs upon exposure to nickel sulfate in 2 out of 3 donors, suggesting that indeed circulating nickel ions are able to trigger migration of LCs.

Due to complexity and costs, unexposed cultures were not included in the experiments, and therefore, the effect of vehicle alone could not be determined. However, tissue histology of exposed cultures (either vehicle or NiSO_4_) compared to unexposed cultures before placement on chips showed no significant differences. Unfortunately, it is not possible to compare LDH, lactate, or glucose levels in these unexposed cultures before application onto the chip due to different volumes in culture supernatants, and dynamic co-culture could not be compared with static monocultures.

Sample size was a limiting factor in our current study. Currently, real-time sensors and probes are being developed to automatically analyze LDH, glucose, and lactate production in organ-on-chip models. This will significantly reduce the sample size and number of replicates within a single experiment making larger scale implementation more feasible in the future. Until these are fully implemented, standardized quality controls with one replicate representing the batch quality could be included for LDH, glucose, and lactate production. For each test condition, since the 3 quality control markers correlate very closely, only one could be selected to reduce the amount of culture supernatant. Our choice would be for LDH as in addition to being a quality control, it is also an assessment of cytotoxicity after compound exposure. Furthermore, the development of multiplexed panels to assess cytokines and metabolites (protein and RNA) would decrease the number of replicates and the amount of tissue required in organ-on-chip models.

Here, we made use of multi-organ complex organotypic culture models with immune cells embedded in the epidermis of the skin construct. Previously, we have shown that the immune competent RHS-LC allows for the investigation of KE-1, 2, and 3 of the AOP for allergic contact dermatitis after topical application of a sensitizer (Rodrigues Neves et al., 2020), and here, we show that this model also responds to a systemic exposure via topical exposure to a distant organ when incorporated into a microfluidics device. In order to also study KE-4 (interaction of antigen presenting cells with T cells), one could consider introducing (donor matched) T cells into the circulation of the microfluidics device. To further enhance these models and to better mimic an immunological competent skin, other cell types could be introduced into the described culture model such as dermal DCs in the RHS-LC.

Investigating systemic toxicity *in vitro* is still a huge challenge. Here, a multi-organ-on-chip approach is presented as an experimental study of topical exposure of oral mucosa to metals which are known to activate the immune system and in turn may result in skin inflammation. Although the performed study is technically challenging and considerable experience is required to build the constructs, we were able to generate reproducible results with a low intra-experimental variation. The presented multi-organ-on-chip setting can be used to further study systemic toxicity and immune cell activation, and can provide a framework for studying other organoids in the future.

## Data Availability

The raw data supporting the conclusions of this article will be made available by the authors, without undue reservation.
